# Administration of Human Derived Upper gut Commensal *Prevotella histicola* delays the onset of type 1 diabetes in NOD mice

**DOI:** 10.1186/s12866-021-02406-9

**Published:** 2022-01-04

**Authors:** Eric Marietta, Irina Horwath, Stephanie Meyer, Shahryar Khaleghi-Rostamkolaei, Eric Norman, David Luckey, Baskar Balakrishnan, Ashutosh Mangalam, Rok Seon Choung, Veena Taneja, Joseph A. Murray

**Affiliations:** 1 Department of Gastroenterology and Hepatology (Celiac Disease), Mayo Clinic, Rochester, MN USA; 2 Department of Immunology, Mayo Clinic, Rochester, MN USA; 3 Department of Dermatology, Mayo Clinic, Rochester, MN USA; 4 Department of Immunology, University of Iowa, Iowa City, Iowa USA

**Keywords:** Diabetes, prevotella, Histicola, Microbiome, Non-obese

## Abstract

**Background:**

Type 1 diabetes (T1D) is an autoimmune disease that is increasing in prevalence worldwide. One of the contributing factors to the pathogenesis of T1D is the composition of the intestinal microbiota, as has been demonstrated. in T1D patients, with some studies demonstrating a deficiency in their levels of *Prevotella*. We have isolated a strain of *Prevotella histicola* from a duodenal biopsy that has anti-inflammatory properties, and in addition, alters the development of autoimmune diseases in mouse models. Therefore, our hypothesis is that the oral administration of *P. histicola* might delay the development of T1D in the non-obese diabetic (NOD) mice. To assess this, we used the following materials and methods. Female NOD mice (ages 5–8 weeks) were administered every other day *P. histicola* that was cultured in-house. Blood glucose levels were measured every other week. Mice were sacrificed at various time points for histopathological analysis of the pancreas. Modulation of immune response by the commensal was tested by analyzing regulatory T-cells and NKp46+ cells using flow cytometry and intestinal cytokine mRNA transcript levels using quantitative RT-PCR. For microbial composition, 16 s rRNA gene analysis was conducted on stool samples collected at various time points.

**Results:**

Administration of *P. histicola* in NOD mice delayed the onset of T1D. Beta diversity in the fecal microbiomes demonstrated that the microbial composition of the mice administered *P. histicola* was different from those that were not treated. Treatment with *P. histicola* led to a significant increase in regulatory T cells with a concomitant decrease in NKp46+ cells in the pancreatic lymph nodes as compared to the untreated group after 5 weeks of treatment.

**Conclusions:**

These observations suggest that *P. histicola* treatment delayed onset of diabetes by increasing the levels of regulatory T cells in the pancreatic lymph nodes. This preliminary work supports the rationale that enteral exposure to a non pathogenic commensal *P. histicola* be tested as a future therapy for T1D.

**Supplementary Information:**

The online version contains supplementary material available at 10.1186/s12866-021-02406-9.

## Background

Type 1 diabetes (T1D) has been increasing in prevalence in the last couple of decades [1–3]. Studies have shown that the intestinal microbiome of T1D patients is altered from healthy controls (dysbiosis) [4–6]. One study demonstrated that placing non-obese diabetic (NOD) mice, a mouse model of T1D, into a germ free condition inhibited the development of hyperglycemia in these mice [7]. In a prior study, we demonstrated that altering the composition of the microbiome through dietary changes can also result in a low incidence of hyperglycemia in NOD mice [8]. Another study found that altering the composition of the intestinal microbiome by administering antibiotics actually accelerated the onset and increased the level of severity of hyperglycemia in NOD mice [9]. These studies clearly suggest that the intestinal microbiome has a significant impact upon the development of hyperglycemia in NOD mice. However, results with humans are not as clear. A number of epidemiological studies have shown that administering antibiotics for infections during childhood did not increase the risk for T1D in genetically susceptible individuals [10, 11], albeit the study by Mikkelsen et al. demonstrated that administering antibiotics 5 times or more in infancy increased the risk of T1D [11]. Genetically susceptible children born by Cesarean section (broad spectrum antibiotic given to the mother) are also at greater risk of developing T1D than one that is born vaginally [12]. In addition, one study reported that the microbiomes of a child born by Cesarean section had bacteria that were from the mother’s skin and oral mucosa, which was dominated by *Staphylococcus, Corynebacterium*, and *Propionibacterium*; however, children born vaginally had microbiomes composed primarily of bacteria associated with the mother’s vagina, dominated by *Lactobacillus, Prevotella*, and *Sneathia* [13]. These data suggest that while some microbiota can inhibit the development of T1D, others such as *Lactobacillus casei* to NOD mice leads to the suppression of diabetes [14]. A clinical trial testing the efficacy of *Lactobacillus Johnsonii* to alter immunological reactions in adult patients with T1D is expected to be concluded in July of 2020 (*NCT03961347****)***. Recently we identifieded a novel strain of *Prevotella histicola (P. histicola*) with anti inflammatory properties that effectively inhibited collagen induced arthritis (CIA) and experimental autoimmune encephalomyelitis (EAE) in humanized mice [15, 16]. In addition, some studies have demonstrated that T1D patients have lower levels of *Prevotella* species as compared to healthy controls [17, 18]. Other publications have shown that NOD mice destined to develop T1D also have decreased levels of *Prevotella* [19, 20]. Because of its effectiveness in inhibiting the mouse models of rheumatoid arthritis (CIA) and multiple sclerosis (EAE), we tested our strain of *P. histicola* for its ability to delay or inhibit the development of T1D in NOD mice.

## Results

### Early treatment with P.histicola delays the onset of T1D

To determine if the long term administration of *P. histicola* did affect the onset of T1D in NOD mice, 5–8 week old female NOD mice (Jackson Laboratories) were allowed to acclimate for 1 week, and then given live *P. histicola* every other day. As figure one demonstrates, orally administering *P. histicola* significantly delayed the onset of T1D. The initial onset for the control mice that were not given *P. histicola* was 14 weeks of age, and the median onset age for the untreated group was 21 weeks of age. In comparison, initial onset for the mice treated with *P. histicola* was 17 weeks of age and the median onset for the treated group was greater than 25 weeks of age. *P histicola* also delayed the onset as compared to additional controls of water alone (sham) and bacterial media alone (Supplementary Fig. 1).

### Pancreatic histopathology

**Fig. 1:**
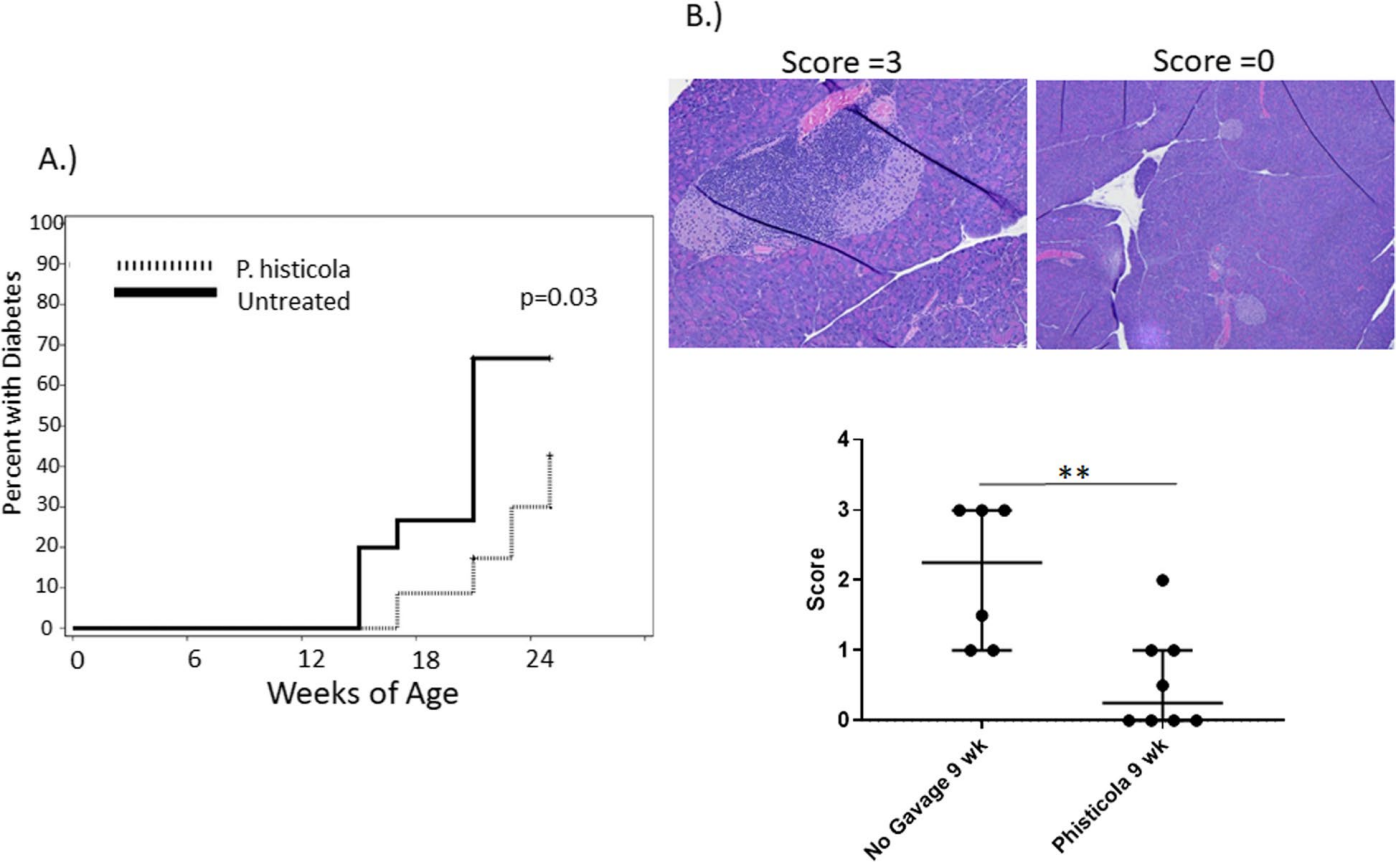
**A**. Incidence of Diabetes. Five to – eight week old female NOD mice on a regular chow were administered *P. histicola* (dashed line, *n* = 23) or not (solid line, *n* = 15). Initial onset for the untreated group was 14 weeks of age and 16 weeks of age for the *P. histicola* treated group. The mean onset for the untreated was 21 weeks of age, and over 25 weeks of age for the treated group, which was significant (*p* = 0.03 Gray K-Sample). B . H&E staining of Pancreas. Hematoxylin and eosin stained pancreata from female NOD mice administered *P. histicola* for 3 weeks (9 weeks of age) were evaluated for inflammation on a score from 0 to 3. *P. histicola* treatment significantly decreased the inflammation of the pancreas score from a median of 2 to 1.5 (*p* < 0.01)

**Fig. 2:**
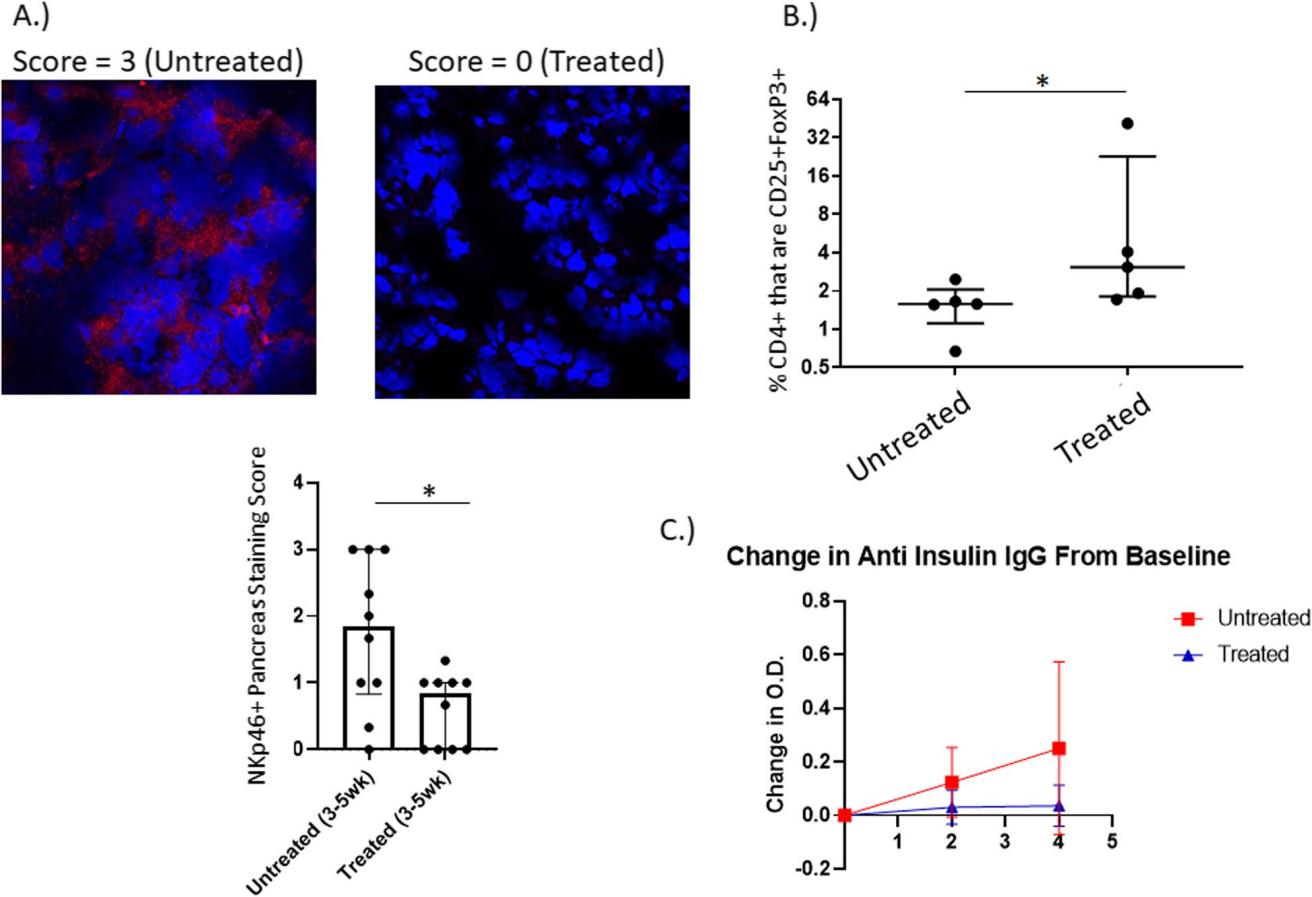
**A**. Immunohistochemistry of Pancreas. Cryosections of pancreata from NOD mice that were treated with *P histicola* for 3–5 weeks were stained for the presence of NKp46 and scored for intensity on a scale from 0 to 3. Treatment with *P. histicola* significantly decreased the intensity for NKp46 staining (*p* < 0.05). **B**. Regulatory T cells. NOD mice were treated with *P. histicola* (Treated) for between 3 and 5 weeks, and pancreatic lymph nodes extracted. % of CD4+ cells that are CD25%FoxP3+ cells significantly increased in the group treated with *P. histicola* (*p* < 0.05). Percentages were obtained by first gating on CD4+ cells, then generating dot plots of CD25 vs FoxP3. **C**. Levels of anti insulin IgG. Circulating levels of anti insulin IgG were evaluated at baseline, 2 weeks of treatment with *P. histicola*, and 4 weeks of treatement with *P. histicola*. The change in O. D, from baseline is plotted above and the mice that were treated with *P. histicola* (*n* = 10) had much less of an increase in anti insulin IgG than the untreated mice (*n* = 10)

At 21 weeks of age, 14% of untreated mice had islet cells in the pancreas as determined by hematoxylin and eosin (H&E) staining, while 85% of mice in the treated group had islet cells remaining in their pancreas (Data not shown). These results demonstrate that *P. histicola* does have a protective effect upon the development of T1D. However, this was not complete protection, as the pancreatic *B* islet cells of the *P. histicola* treated mice at 21 weeks of age had become infiltrated. In order to determine if there was a difference in the pathology of the pancreas at earlier time points, mice were sacrificed at different time points of treatment with *P. histicola*: 13 weeks of age (7 weeks of treatment), 11 weeks of age (5 weeks of treatment), and 9 weeks of age (3 weeks of treatment). There was a significant difference in the level of cellular infiltration of the pancreas after 3 weeks of *P histicola* treatment (*p* < 0.01), but by 5 weeks of treatment, there was no significant difference between the two groups in the level of infiltration (Fig. 1 and supplementary Fig. 2). Immunohistochemistry staining of the pancreata of the NOD mice treated between 3 weeks and 5 weeks post treatment (9 weeks and 11 weeks of age respectively) revealed no difference in the number of FoxP3 + cells, CD4+ cells, or B220+ cells (supplementary Fig. 3). Additionally, no difference was found in the number of CD8+ cells after 5 weeks of treatment (supplementary Fig. 3). However, there was a significant reduction of NKp46+ cells in the pancreas of the NOD mice treated with *P histicola* for between 3 and 5 weeks (Fig. 2) of treatment. NKp46 is an activating receptor that is expressed by NK (natural killer) cells and innate lymphoid cells type 1 (ILC1) [21, 22] and has been demonstrated to be essential for the development of T1D, as the NKp46 ligand is expressed by pancreatic β islet cells [23]. This would suggest that in the NOD mouse, administration of *P. histicola* results in fewer NKp46+ cells in the pancreas.

### Administration of P.histicola impacts systemic immune responses

**Fig. 3:**
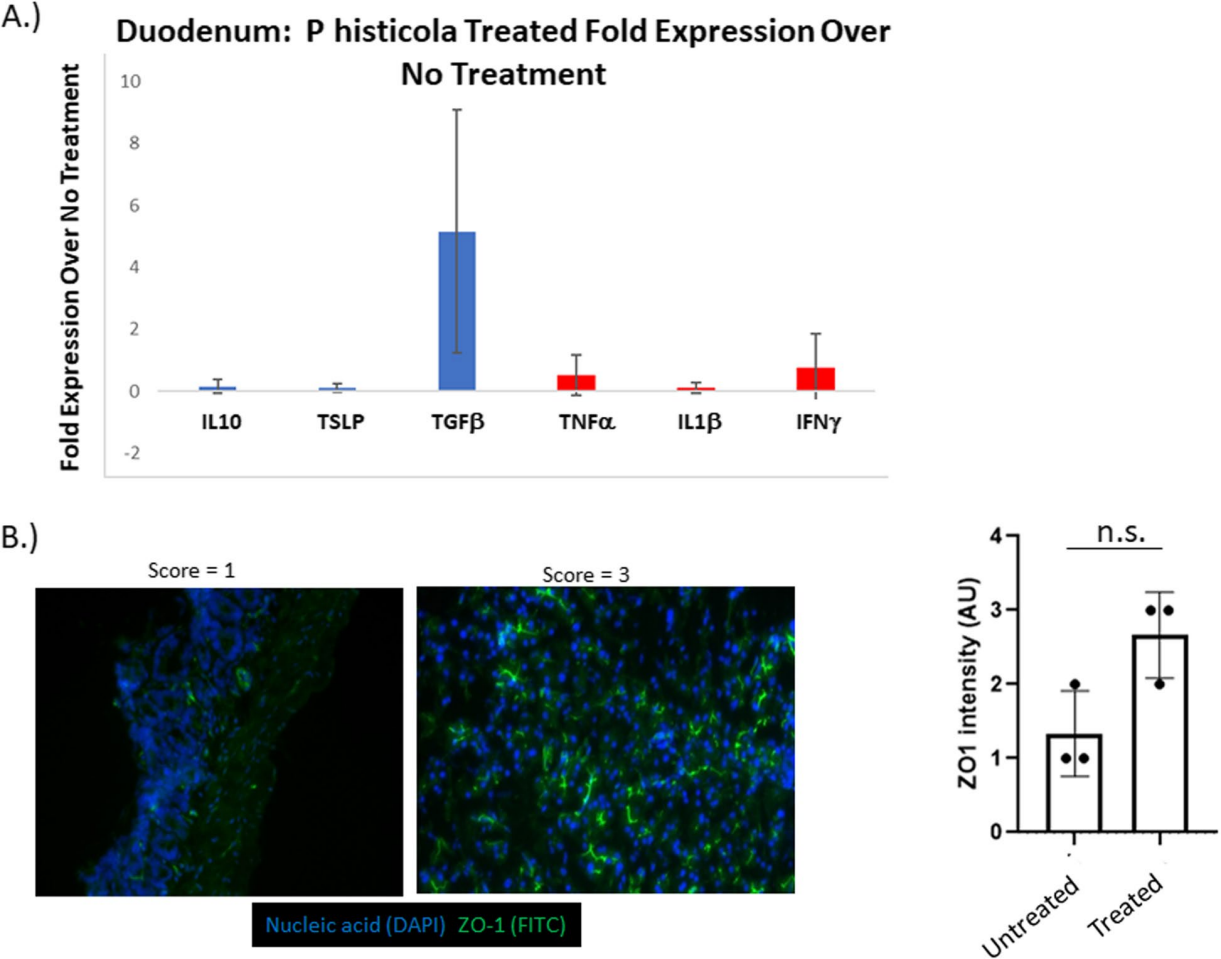
**A**. Intestinal cytokine mRNA. Duodenal segments from NOD mice 18 h after an oral treatment with *P. histicola* (*n* = 4) were evaluated for the fold expression of each cytokine over no treatment (*n* = 4). TGFβ had almost five fold increase with *P histicola* treatment. **B**. Zo1 Intestinal Expression. 18 h after treatment with *P. histicola*, the duodenum was extracted and stained for ZO-1. Intensity scores were on a scale of 0–3. The treated mice (*n* = 3) had a non-significant increase in ZO-1 expression over untreated (*n* = 3)

FACS analysis of the mesenteric lymph nodes, Peyer’s patches, pancreatic lymph nodes, and spleen were done on mice treated with *P histicola* for between 3 and 5 weeks. Regulatory T cells (CD4+ CD25+ Foxp3+) made up a greater proportion of the CD4+ cells in the mice that were treated with the *P. histicola*, and was significantly greater in the pancreatic lymph nodes (Fig. 2). Evaluation of anti insulin IgG demonstrated that the administration of *P. histicola* led to less of an increase in circulating anti insulin IgG after 28 days (4 weeks) than in the untreated NOD mice (Fig. 2), even though there was no statistically significant difference. Analysis of duodenal cytokine production 18 h after gavaging with *P. histicola* demonstrated that there was an over five fold increase in the expression of TGFβ transcripts in *P. histicola* treated mice using untreated mice as the control group in the ΔΔ Ct calculation (Fig. 3). In contrast, the inflammatory cytokines, IL1β, IFNγ, and TNFα did not increase in the duodena from *P. histicola* treated NOD mice (Fig. 3). *P histicola* treatment increased expression of ZO-1, a tight junction protein, in the duodena of NOD mice, as shown in Fig. 3, albeit this was not statistically significant.

### Microbiome analyses

**Fig. 4:**
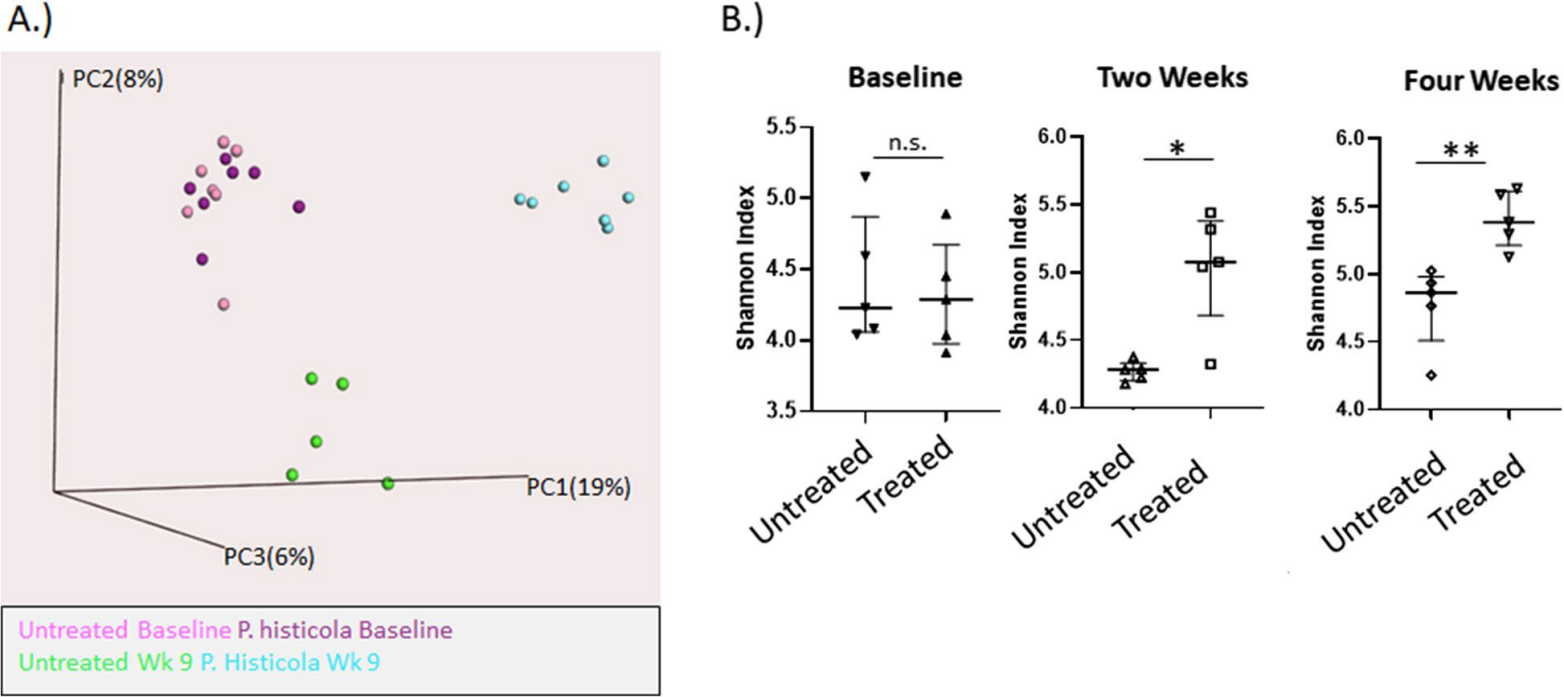
**A**. Beta Diversity Plot of Fecal Microbiomes. Stool was collected at baseline and 9 weeks after administering *P. histicola*. Beta diversity was depicted in a 3 dimensional principal coordinate plot. The baseline of the untreated mice are depicted in pink, the baseline of the *P histicola* treated mice is depicted in lavender, the 9 week untreated group is green, and the 9 week *P. histicola* group is blue. ***B****. Alpha* Diversity Plot. Shannon Index is plotted for fecal microbiomes from baseline, 2 weeks, and 4 weeks of *P histicola* treatment along with corresponding untreated groups. There is an increasing trend of significant difference with time, such that the treated group is significantly higher than untreated at 2 weeks (*p* < 0.05). At 4 weeks after treatment the difference between the untreated and treated groups is greater (*p* < 0.01). *n* = 5 mice for untreated and n = 5 mice for *P histicola* treated

**Fig. 5:**
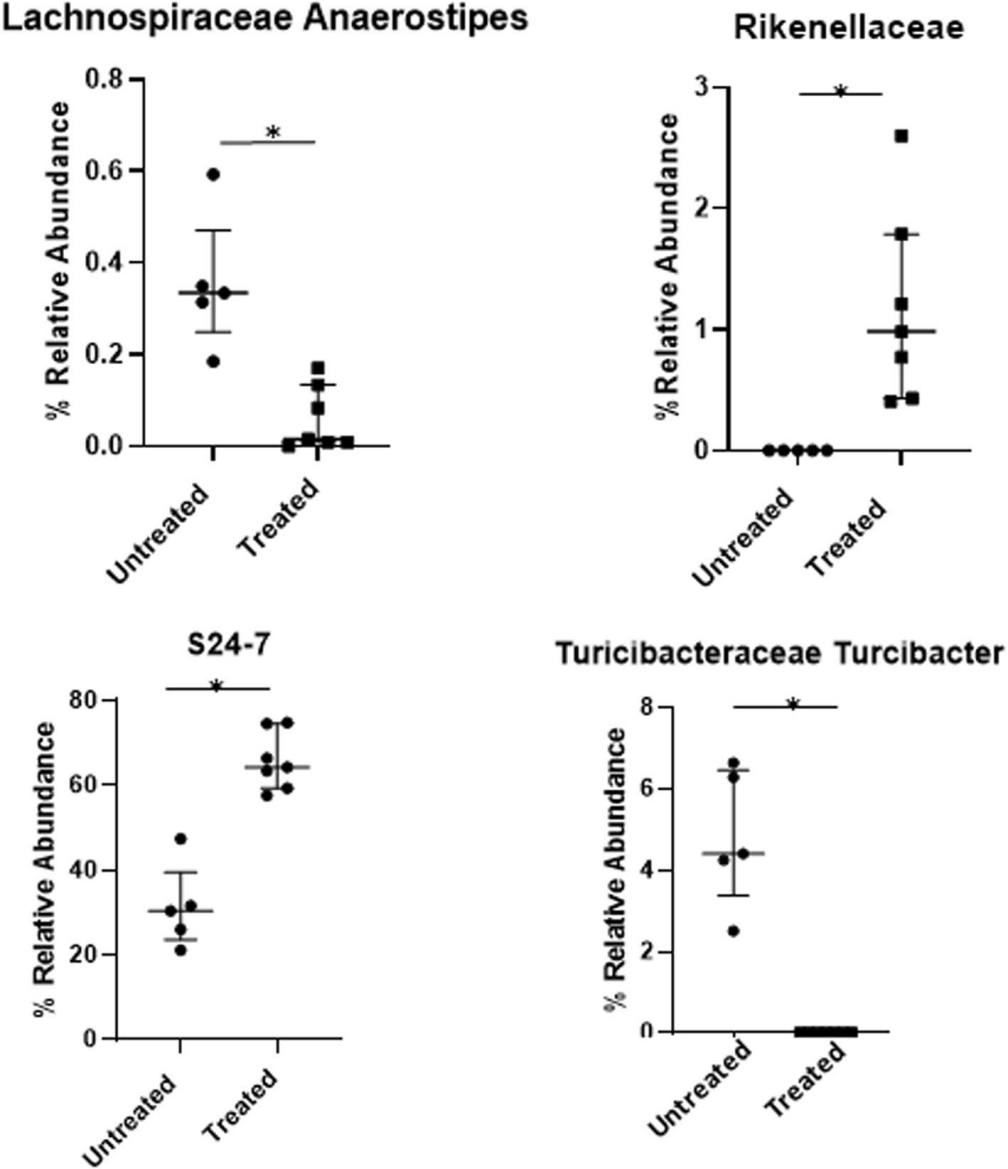
**A**. Increases in bacterial groups with *P histicola* treatment. The taxonomy of the fecal microbiomes at 9 weeks were compared and those bacterial groups that significantly increased were S24–7 and *Rikenellaceae* (*p* < 0.01 each). **B**. Decreases in bacterial groups with *P histicola* treatment. The bacterial groups that were significantly decreased with *P. histicola* treatment were Clostridium and the genus *Staphylococcaceae Staphylococcus* (*p* < 0.01 each). *Turcibacteraceae Turcibacter* and *Lachnospiraceae Ruminococcus* were also significantly decreased (*p* < 0.01 and *p* < 0.05, respectively)

In order to determine what the changes were to the intestinal microbiomes of the NOD mice, analyses and comparisons of fecal samples were done using 16 s rRNA gene analysis. A beta diversity principal component plot demonstrates that the composition of the microbiome clearly changes after 9 weeks of administration of *P. histicola* (Fig. 4). The fecal microbiomes of the untreated mice also shifted from baseline, and this was distinct and separate from the fecal microbiomes of the mice treated with *P. histicola* for 9 weeks. Alpha diversity, as measured by Chao1, did not differ significantly between the untreated and *P. histicola* treated mice at any of the time points evaluated (data not shown). However, Alpha diversity, as evaluated by Shannon Index, demonstrated significant increases with 2 weeks of administering *P. histicola* along with a continued increase after 4 weeks of administering *P. histicola*, whereas the fecal microbiomes of the untreated mice did not change over the same time period (Fig. 4). The bacterial family S24–7 showed the most significant change (from 30% relative abundance of OTUs to 65% relative abundance of OTUs) and was the most abundant bacterial family in the mice (Fig. 5). A recent publication has suggested renaming S24–7 as *Muribaculaceae* [24], and found that the *Muribaculaceae* family was dominant in rodents, but was also found in other animals, including humans. This bacterium, S24–7, was associated with protection from diabetes in NOD mice [19]. *Rikenellaceae* was also increased with the administration of *P. histicola*, although the relative abundance was only 1% after the administration of *P. histicola*; however, a previous study has demonstrated that T1D in NOD mice is associated with decreased levels of *Rikenellaceae* [25]. The administration of *P. histicola* also inhibited the increase of *Staphylococceae Staphylococcus* and the *Clostridium* and *Turcibacter* families that was observed in the untreated control mice (Fig. 5).

## Discussion

Treatment of NOD mice, a mouse model of T1D, with *P. histicola* effectively delays the onset of diabetes in these mice. Our results indicate that the administration of *P. histicola* to the NOD mice results in increased levels of regulatory T cells in the pancreatic draining lymph nodes. This hypothesis is supported by another study that found that regulatory T cells can traffic from the gut to the pancreatic lymph nodes [26]. Additionally, administering *P. histicola* significantly reduces the overall cellular infiltrate in the pancreatic β islets. When looking at immune cells present in the pancreas (CD3+, CD4+, CD8+, B220+, NKp46+), only those that express NKp46, are the cells that are significantly reduced by the administration of *P. histicola*. This would suggest that the administration of *P. histicola* exerts an anti inflammatory response in the intestines of the NOD mice, which then downregulates the extraintestinal inflammatory autoimmune response occuring in the pancreas of the NOD mice. We have previously shown that *P. histicola* colonizes the duodenum in mice and leads to an increase in intestinal Tregulatory cells and CD11b + myeloid suppressors [15, 27].

Data presented in this manuscript with NOD mice also shows that the administration of *P. histicola* increases the alpha diversity of the intestinal microbiomes of the mice. This result is similar to our findings in the mouse models of RA and MS. All of which suggest that the administration of *P. histicola* will improve the health of the intestinal microbiomes of the mice.

Of great interest is that the administration of *P. histicola* does not cause the same changes in specific bacterial groups as observed in the mouse model of MS. This is probably due to differences in the genetics of the mice, which results in different compositions of bacterial groups in the mice after weaning, and potentially before they are weaned as well. One of our previous publications demonstrated that a 3 amino acid difference in HLADR4 (0401 vs 0402) molecules resulted in differences in the intestinal microbiomes of the mice [28]. Therefore, it is quite reasonable to assume that genetic differences as great as the NOD genetic background from those of the humanized mice (HLA-DR4 and HLADR3) in the CIA and EAE studies would result in different baseline intestinal microbiomes before administering *P. histicola* and consequentially changes in intestinal bacterial groups after the administration of *P. histicola* differ between the models*.* Because of this then, it is unlikely that specific changes in the intestinal microbiome are exerting the anti-inflammatory responses in the intestinal epithelium, and are instead due to the intestinal epithelium responding to *P. histicola* or associated bacterial products. This is supported by an experiment in one of our previous observations in which the administration of antibiotics to deplete the microbiome of most bacteria, followed by the administration of *P.histicola* still inhibited the development of EAE in the mice [16]. A similar experiment with these NOD mice is not feasible, as studies have shown that administering antibiotics to NOD mice itself exacerbates the development of hyperglycemia [9, 25].

However, there are some limitations to this study. One limitation is that the study did not evaluate the mice beyond 25 weeks of age. Another limitation is that additional analyses beyond the incidence of diabetes was not done on the NOD mice gavaged with water alone (sham) and gavage with bacterial media. We also did not address the means by which *P histicola* can increase the number of Tregs. One example by which this may occur would be through CD11b + CD11c- antigen presenting cells (APCs) as demonstrated by Richer et al. [29] and by our previous studies [15].

## Conclusions

In this study, we have shown that *P. histicola* effectively delays the onset of diabetes in NOD mice. From the three different mouse models of autoimmune disease (RA, MS, and now with this manuscript, T1D), we have observed that the commensal, *P. histicola*, will exert anti-inflammatory immune responses in the intestine. This intestinal anti-inflammatory immune response can then down regulate inflammatory autoimmune responses in different tissues throughout the animal. Overall then, these three studies support the premise that the administration of *P. histicola* could benefit patients with autoimmune diseases or who are at high risk of developing these autoimmune diseases.

## Methods

### Aim

To determine if the oral administration of *P. histicola* might delay the development of T1D in the non-obese diabetic (NOD) mice.

### NOD mice

Female NOD Shi/Ltj mice were purchased from Jackson Laboratories (Bar Harbor, Maine), aged between 5 weeks and 8 weeks of age. All mice were conventionally housed and maintained on a regular irradiated LabDiet rodent chow.

### Method of euthanasia

All mice used in this study were humanely euthanized by CO_2_ inhalation as approved and directed by the Mayo Clinic IACUC.

### Blood glucose measurement

Glucose levels were monitored every two weeks using a glucometer (Contour, Parsippany, NJ). Diabetes was determined when two consecutive blood glucose measurements were 250 mg/dL.

### Bacterial culture and administration

*P. histicola* (MCI 001) was cultured using a tryptic soy broth (TSB) media under strictly anaerobic condition using a Bactron anaerobic chamber (SHELDON MANUFACTURING, INC, USA). The gas mixture was N2 (85–90%), H2 (5%) and CO2 (5–10%). The culture conditions were 37 °C for 48 h, with a pH of 7.2 ± 0.2. The purity of the bacterial culture was frequently checked, as published previously [30]. For administration to the mice by oral gavage, the bacterial concentration was 10^9^ CFU mL^-1,^ where each mouse received 100 μL of culture containing approximately 10^8^ CFU of the bacterium. Administration of the prepared *P. histicola* culture to the mice by gavage was done every other day, as previously described [15].

### Anti insulin IgG

The measurement of anti insulin IgG by ELISA was performed as described before [8].

### Histopathology

Sections of pancreas were formalin fixed and snap frozen in OCT (optimal cutting temperature compound from Tissue Tek, Sakura). Duodenal sections were taken from NOD mice 18 h after treatment with *P. histicola* and snap frozen. Formalin fixed pancreatic tissue was paraffin embedded and one section stained with hematoxylin and eosin and scored for cellular infiltration of all the islets in the section, based on a score of 0–3. We did not isolate the islets from the pancreas in order to evaluate 35 different islets from each mouse pancreas in order to do a highly rigorous analysis of the islets for insulitis and cell markers by immunhistoochemistry as was done by Koide et al. and Inoue et al. [31, 32].

For immunohistochemistry of frozen tissue (IHF), frozen OCT blocks were cryosectioned and one to two sequential sections stained with the following fluorochrome conjugated antibodies (Fitc anti mouse CD3, Fitc anti mouse CD4, Percp anti mouse CD8, APC anti mouse FoxP3, and Fitc anti mouse B220). All conjugated antibodies were purchased from Becton Dickinson. Unconjugated anti mouse NKp46 was purchased from US Biological, and unconjugated anti mouse ZO-1 was purchased from Invitrogen. The secondary antibody for anti mouse ZO-1 was Fitc anti rabbit IgG and the secondary for anti NKp46 was AF594 anti rabbit IgG, both of which were purchased from Jackson ImmunoResearch. All frozen immunohistochemistry was evaluated on a confocal laser microscope (Zeiss LSM 780), analyzed using Zen Black software, and then scored on a scale of 0–3, similar to the H&E staining.

### Regulatory T cells by FACs analysis

After sacrifice, the pancreatic draining lymph nodes, the mesenteric lymph nodes, Peyer’s patches, and splenocytes were extracted and stained with, Fitc anti mouse CD25, PE anti mouse CD4, and APC anti FoxP3 and then analyzed by flow cytometry. All antibodies were purchased from Becton Dickinson. Analysis was done using FlowJo software. For the analysis of Tregs, cells were gated first on CD4+ cells, then the percentages of CD25 + FoxP3+ cells were obtained (Supplementary Fig. 4).

### Cytokine transcription

Segments of duodenum were extracted from NOD mice that were sacrificed 18 h after treatment with *P. histicola* and snap frozen. RNA was extracted using the RNAEasy Plus mini kit from Qiagen. cDNA was generated using a Superscript III reverse transcriptase kit from Invitrogen (Thermo Fisher), and semi-quantitative RTPCR done using Sybr Green master mix from Life Technologies (Thermo Fisher) on an ABI-ViiA-7 RT-PCR machine by Applied BioSystems. The specific primers used are previously published [33].

### 16 s microbiome

Fresh stool pellets were collected at the start of the study (baseline) and periodically throughout the experiments. Fecal DNA was extracted using the Mo-Bio PowerSoil extraction kit (Qiagen) with a bead beating step as well as an incubation period for optimal yield. Reverse primer barcodes were synthesized by Integrated DNA Technologies (IDT, Coralville, Iowa) and ordered as HPLC purified oligos, targeting V3-V5 regions of the 16 s ribosomal RNA gene. DNA was amplified using 357 Forward primer and reverse primer barcodes specified by Illumina as done previously [8]. Extracted DNA underwent 25 cycles on the PCR and both amplicons and generated libraries were quantitated with a Qubit 3.0 Fluorometer (Thermo Fisher). Libraries were sequenced using Illumina MiSeq machines at Mayo Clinic and at the University of MN. All sequencing files were run through the Tornado pipeline at Mayo Clinic [34].

### Statistical analysis

Statistical analyses were done using Excel, SAS, and GraphPad Prism.

## Supplementary Information

**Additional file 1: Supplementary Figure S1:** Incidence of diabetes. Additional controls of water alone (sham gavage *n*=7 ) and bacterial media alone (n=7) are included. The delayed onset by *P. histicola* was significant (*p*<0.0001 Gray K-Sample)

**Additional file 2: Supplementary Figure S2**: H&E staining of Pancreas at 5 weeks and 7 weeks of treatment. Hematoxylin and eosin stained pancreata from female NOD mice administered *P. histicola* for 5 weeks (11 weeks of age) and 7 weeks (13 weeks of age) were evaluated for inflammation on a score from 0 to 3. *P. histicola* treatment did not significantly decrease the inflammation of the pancreas score in either the 5 weeks of treatment (*p*=0.4) or the 7 weeks of treatment (*p*=0.5).

**Additional file 3: Supplementary Figure S3**: Immunofluorescent IHC of the pancreas for CD4, FoxP3, CD8, and B220. Pancreata from female NOD mice administered *P. histicola* for 3 weeks (9 weeks of age) were evaluated for CD4, FoxP3, and B220, and mice administered *P. histicola* for 5 weeks (11 weeks of age) were evaluated for CD4, FoxP3, CD8, and B220.

**Additional file 4: Supplementary Figure S4:** Flow cytometry gating for regulatory T cells. Using data gathered from flow cytometry analysis and FloJo software, cells were first gated on CD4+ cells. A dot plot with CD25 and Foxp3 on the axes was then generated.

## Data Availability

The datasets generated in the current study are available in the NCBI BioProject database, accession # PRJNA785662.

## Abbreviations

T1D: Type 1 diabetes
NOD: Non-obeses diabetic
CIA: Collagen induced arthritis
EAE: Experimental encephalomyelitis
*P. histicola*: *Prevotella histicola*
H&E: Hematoxylin and eosin
NK: Natural killer
FACS: Fluorescence activated cell sorting
OTU: Operational taxonomic unit
TSB: Tryptic soy broth
CFU: Colony forming unit
IHF: Immunohistochemistry frozen
OCT: Optimal cutting temperature compound
HPLC: High performance liquid chromatography
