# Supplementary figures and images for: Administration of Human Derived Upper gut Commensal Prevotella histicola delays the onset of type 1 diabetes in NOD mice
Source: BMC Microbiol. 2022 Jan 4;22:8. doi: 10.1186/s12866-021-02406-9 (PMC8729070; doi:10.1186/s12866-021-02406-9)

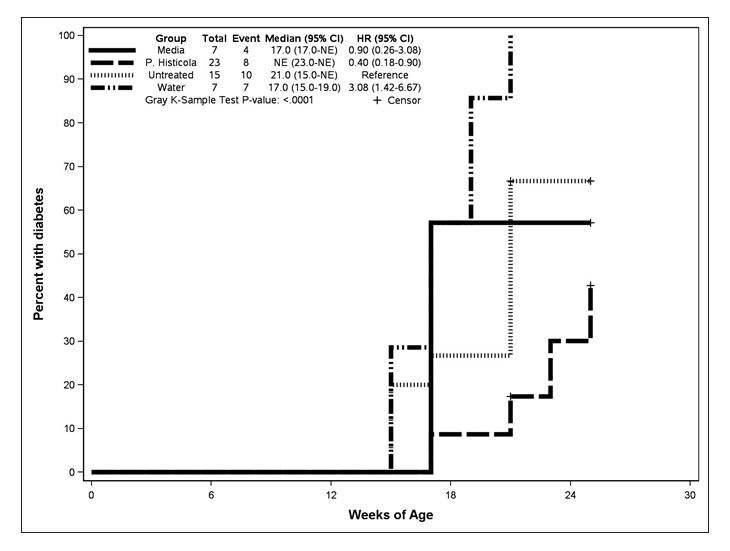

Supplement: Supplementary file 1 — Additional file 1: Supplementary Figure S1: Incidence of diabetes. Additional controls of water alone (sham gavage n=7 ) and bacterial media alone (n=7) are included. The delayed onset by P. histicola was significant (p<0.0001 Gray K-Sample) [file 12866_2021_2406_MOESM1_ESM.tif]

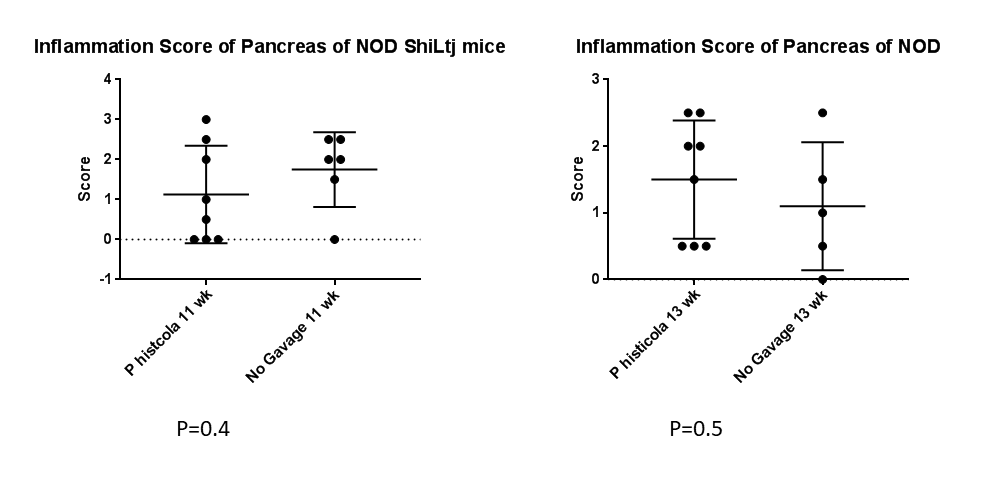

Supplement: Supplementary file 2 — Additional file 2: Supplementary Figure S2: H&E staining of Pancreas at 5 weeks and 7 weeks of treatment. Hematoxylin and eosin stained pancreata from female NOD mice administered P. histicola for 5 weeks (11 weeks of age) and 7 weeks (13 weeks of age) were evaluated for inflammation on a score from 0 to 3. P. histicola treatment did not significantly decrease the inflammation of the pancreas score in either the 5 weeks of treatment (p=0.4) or the 7 weeks of treatment (p=0.5). [file 12866_2021_2406_MOESM2_ESM.tif]

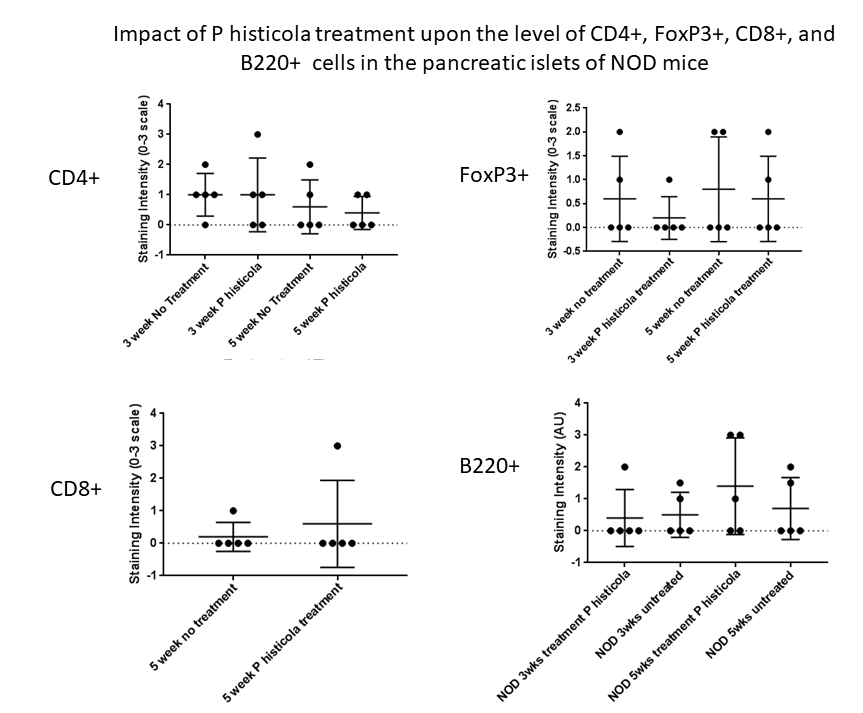

Supplement: Supplementary file 3 — Additional file 3: Supplementary Figure S3: Immunofluorescent IHC of the pancreas for CD4, FoxP3, CD8, and B220. Pancreata from female NOD mice administered P. histicola for 3 weeks (9 weeks of age) were evaluated for CD4, FoxP3, and B220, and mice administered P. histicola for 5 weeks (11 weeks of age) were evaluated for CD4, FoxP3, CD8, and B220. [file 12866_2021_2406_MOESM3_ESM.tif]

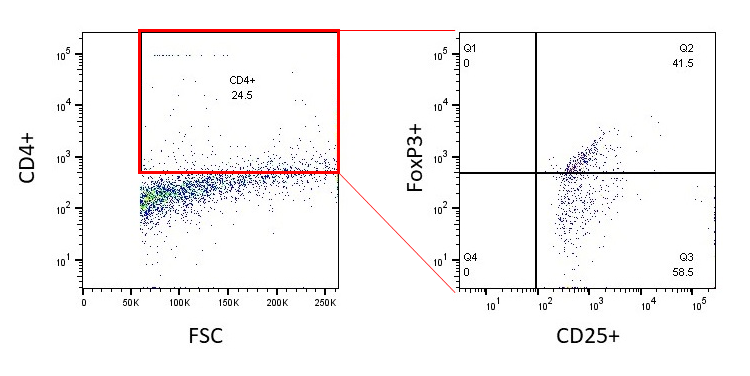

Supplement: Supplementary file 4 — Additional file 4: Supplementary Figure S4: Flow cytometry gating for regulatory T cells. Using data gathered from flow cytometry analysis and FloJo software, cells were first gated on CD4+ cells. A dot plot with CD25 and Foxp3 on the axes was then generated. [file 12866_2021_2406_MOESM4_ESM.tif]
